# Analysis of the Volumes of the Posterior Cranial Fossa, Cerebellum, and Herniated Tonsils Using the Stereological Methods in Patients with Chiari Type I Malformation

**DOI:** 10.1100/2012/616934

**Published:** 2012-05-02

**Authors:** Ümit Erkan Vurdem, Niyazi Acer, Tolga Ertekin, Ahmet Savranlar, Mehmet Fatih İnci

**Affiliations:** ^1^Department of Radiology, Kayseri Education and Research Hospital, 38010 Kayseri, Turkey; ^2^Department of Anatomy, Erciyes University School of Medicine, 38039 Kayseri, Turkey; ^3^Department of Radiology, Elazığ Harput State Hospital, 23200 Elazığ, Turkey

## Abstract

*Objective*. The aim of this study was to determine the posterior cranial fossa volume, cerebellar volume, and herniated tonsillar volume in patients with chiari type I malformation and control subjects using stereological methods. *Material and Methods*. These volumes were estimated retrospectively using the Cavalieri principle as a point-counting technique. We used magnetic resonance images taken from 25 control subjects and 30 patients with chiari type I malformation. *Results*. The posterior cranial fossa volume in patients with chiari type I malformation was significantly smaller than the volume in the control subjects (*P* < 0.05). In the chiari type I malformation group, the cerebellar volume was smaller than the control group, but this difference was not statistically significant (*P* > 0.05). In the chiari type I malformation group, the ratio of cerebellar volume to posterior cranial fossa volume was higher than in the control group. We also found a positive correlation between the posterior cranial fossa volume and cerebellar volume for each of the groups (*r* = 0.865, *P* < 0.001). The mean (±SD) herniated tonsillar volume and length were 0.89 ± 0.50 cm^3^ and 9.63 ± 3.37 mm in the chiari type I malformation group, respectively. *Conclusion*. This study has shown that posterior cranial fossa and cerebellum volumes can be measured by stereological methods, and the ratio of these measurements can contribute to the evaluation of chiari type I malformation cases.

## 1. Introduction

The chiari malformations generally describe increasing degrees of hindbrain herniation through the foramen magnum. Chiari type I malformation (CMI) is defined as a herniation of the cerebellar tonsils of at least 5 mm or more through the foramen magnum. Syringomyelia is associated with this condition in 50–75% of cases [1, 2].

The etiology of CMI is unclear and may be multifactorial. It is believed to be congenital, even though those who have it do not usually have symptoms until early childhood or adolescence [3, 4]. Some conditions under which the herniation of the cerebellar tonsils occurs include hydrocephalus and intracranial mass. A generally decreased volume of the posterior cranial fossa is thought to be one of the predisposing factors in some cases [5, 6].

Several studies have attributed this insufficient posterior cranial fossa geometry to embryological defects in the paraxial mesoderm [7–9]. The advent of magnetic resonance imaging (MRI) in recent years has made it possible to make an early and accurate noninvasive diagnosis of CMI in patients whose symptoms might be subtle [10, 11].

There are a few studies which have compared cerebellar volume (CV) and posterior cranial fossa volume (PCFV), but there is no study in the literature that aims to estimate the herniation of the cerebellar tonsillar volume and explain the relationship that each of these volumes has with each other.

The aim of this study was to investigate PCFV, CV, and herniated tonsillar volume in patients with CMI. We compared PCFV and CV between the CMI and control groups using stereological methods with MRI.

## 2. Material and Methods

### 2.1. Control Subjects

We carried out the present study on 25 subjects consisting of 12 females (mean age, 40.25 ± 12.14 years) and 13 males (mean age, 38.30 ± 11.14 years). There was no statistically significant difference between sexes according to age.

The subjects were volunteers, and written informed consent was obtained. Official permission was also obtained from the responsible departments of the university and state hospital administrators. All procedures were fully explained to the subjects. Those who underwent an MRI complaining of headaches with the results showing no cranial or intracranial pathology were chosen as the control group.

### 2.2. Patients

We retrospectively evaluated 70 adult patients who had been treated in our neurosurgical department because of CMI between January 2006 and December 2010. Thirty patients (14 males, mean age 37.0 ± 11.6 years and 16 females mean age 41.9 ± 13.8 years) whose preoperative radiological studies were available served as subjects in this study.

The ages of the male and female groups were statistically matching. To be eligible for the study, each patient was required to have MRI findings consistent with CMI. The requirements for enrollment of the latter patients included having access to all preoperative medical records, radiographic studies, and neurological findings. Patients with neurological deficits attributable to surgery were excluded.

### 2.3. Magnetic Resonance Imaging Protocol

We analyzed the neurologically intact cranial MRIs of all the subjects, and we obtained T1- and T2-weighted sagittal images using a 1.5 Tesla MRI machine (GE Signa, HDI, France). The following parameters were used for the imaging process for T1: TR/TE: 425/17.5, FOV: 20, 1.5-mm slice thickness without gap and matrix 288 × 224. The parameters for T2 were as follows: TR/TE: 7450/102, FOV: 20, 1.5 mm slice thickness without gap, and matrix 384 × 288. Both T1- and T2-weighted images were used for examination of the herniated tonsillar images. We also obtained T2-weighted sagittal images using the following protocol for the examination of the posterior cranial fossa and cerebellar sections: TR/TE: 7450/102, FOV: 26, 5 mm slice thickness without gap, and matrix 384 × 288.

### 2.4. The Margins of the Posterior Cranial Fossa

**Figure 1:**
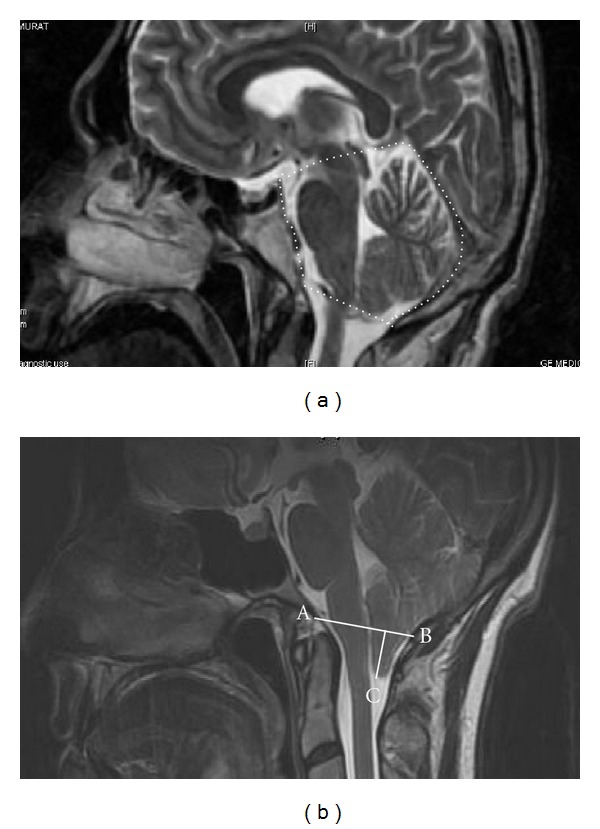
Brain MR midsagittal T2 demonstrating the posterior cranial fossa boundaries (a), the measurement of herniated tonsils from sagittal images with A: basion, B: opisthion, and C: degree of tonsillar herniation measured as the length perpendicular from C to AB (b).

The posterior cranial fossa was defined as the osseous anatomical area with a floor formed by the occipital bone (basioccipital portion of the clivus and supraoccipital portion of the occipital bone up to the insertion of the tentorium cerebella forming the superior boundary of this fossa) and the basisphenoid. The petrous ridges of the temporal bones formed the anterolateral border of this cavity anteriorly to their connection (posterior petroclinoid ligament) to the posterior clinoids [7], (Figure 1(a)).

### 2.5. Measurement of the Herniated Tonsillar Length

The extent of cerebellar herniation was measured from the tips of the cerebellar tonsils to a line drawn between the basion and opisthion (Figure 1(b)).

### 2.6. Cavalieri Estimator

**Figure 2:**
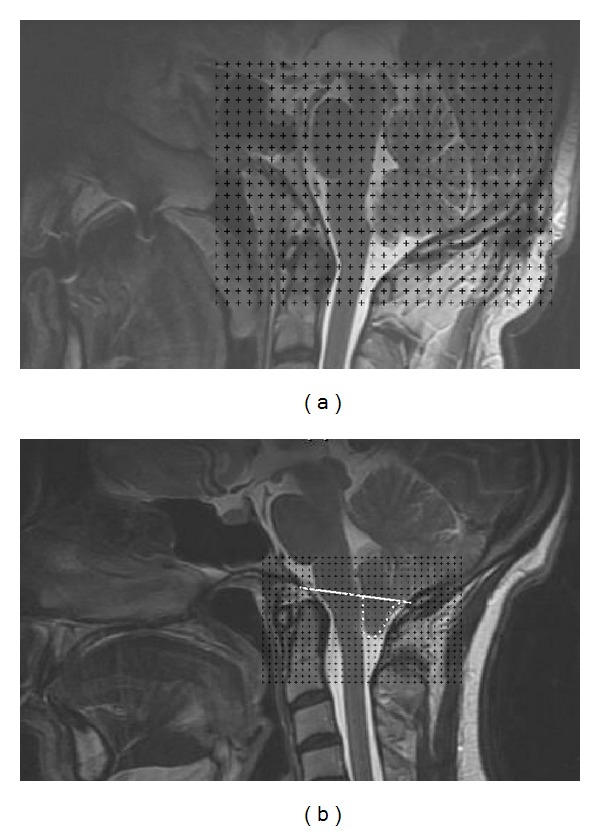
Representative midsagittal MRI for the cranial fossa and cerebellum (a) and herniated tonsils (b) with a grid overlaid for the calculation of volumes using the Cavalieri method.

The MRIs of a section series with 5 mm thickness were used for CV and PCFV estimation. A section series with 1.5 mm thickness was used for tonsillar herniation volume estimation. The images were saved in the computer and the transparent square grid test system with *d* = 0.8 cm between test points was superimposed for the posterior cranial fossa and cerebellum. For herniated tonsillar sections, *d* = 0.25 cm between test points was superimposed randomly covering the entire image frame. The points hitting the cerebellum, posterior cranial fossa, and tonsillar herniation-sectioned surface area were counted for each area (Figures 2(a) and 2(b)). These volumes were estimated using the modified formula for volume estimations of radiological images as shown below [12–14]

(1) $$V=T\times {\left[\frac{SU\times d}{SL}\right]}^{2}\times \sum P.$$

In the formula, “*T*” is the section thickness, “*SU*” the scale unit of the printed film, “*d*” the distance between the test points of the grid, “*SL*” the measured length of the scale printed on the film, and “∑*P*” the total number of points hitting the cut, sectioned surface areas of the cerebellum, posterior cranial fossa, and herniated tonsils.

 According to this volumetric technique, a square grid of test points was positioned on each MRI, and all points that hit were counted.

### 2.7. Volume Fraction Estimation

The volume fraction is used to express the proportion of a phase or component within the whole structure. The volume fraction of an *X* phase within a *Y* reference volume is simply expressed as follows:

(2) $$V_{V}\left(X,Y\right)=\frac{\text{Volume}\;\text{of}\;X\;\text{phase}\;\text{in}\;Y\;\text{reference}\;\text{space}}{\text{Volume}\;\text{of}\;Y\;\text{reference}\;\text{space}}.$$

Volume fraction ranges from 0 to 1 and is often expressed as a percentage [15–17].

 The volume fraction formula with the combination of a point-counting grid can be written as

(3) $$V_{V}\left(X,Y\right)=\frac{\sum P_{X}}{\sum P_{Y}}.$$

In this formula, “∑*P*_*X*_” indicates the number of points hitting the *X* phase and “∑*P*_*Y*_” the number of points hitting the reference space *Y*.

We estimated the volume fraction of the herniated tonsils within the whole cerebellum by means of the following formula:

(4) $$V_{V}\left(\text{Herniation},\text{Cerebellum}\right)=\frac{\sum P_{\text{Herniation}}}{\sum P_{\text{Cerebellum}}}.$$

In this formula, ∑*P*_herniation_ is the total number of points hitting the components of the herniated tonsil, and ∑*P*_cerebellum_ is the total number of points hitting sectioned surface of the cerebellum, including all its parts. 

 The application of the described approaches for the estimation of volume and volume fraction is presented (Table 1):

(5) $$\begin{array}{cc} V\left(\text{Cerebellum}\right) & =T\times {\left[\frac{SU\times d}{SL}\right]}^{2}\times \sum P\\ & =0.5\times \left[\frac{2\times 0.8}{1.95}\right]\times 375=124.55\;\text{c}{\text{m}}^{3},\\ V\left(\text{Herniation}\right) & =T\times {\left[\frac{SU\times d}{SL}\right]}^{2}\times \sum P\\ & =0.15\times \left[\frac{0.2\times 0.25}{0.25}\right]\times 173=1.04\;\text{c}{\text{m}}^{3}, \end{array}$$

(6) $$\begin{array}{cc} \text{CE} & ={\left[\frac{k}{k-1}\left\{\frac{\sum u^{2}}{\sum u\sum u}+\frac{\sum u^{2}}{\sum v\sum v}-2\frac{\sum uv}{\sum u\sum u}\right\}\right]}^{1/2},\\ \text{CE} & ={\left[\frac{21}{21-1}\left\{\frac{8345}{{\left(375\right)}^{2}}+\frac{2207}{{\left(173\right)}^{2}}-2\frac{3964}{\left(375\times 173\right)}\right\}\right]}^{1/2}\\ & =0.10=10\text{\%}. \end{array}$$

The volume fraction of the phase *Y* was estimated as:

(7) $$\begin{array}{cc} & V_{V}\left(\text{Herniation},\text{Cerebellum}\right)\\ & \;\;=\frac{\sum P_{\text{Herniation}}}{\sum P_{\text{Cerebellum}}}=\frac{1.04\;\text{c}{\text{m}}^{3}}{124.55\;\text{c}{\text{m}}^{3}}=0.01=1\text{\%}. \end{array}$$

### 2.8. Error Prediction for Point Counting Technique with Volume Fraction

Accordingly, the efficiency of sampling and the density of grid points were performed as documented in the literature [17, 18]. The coefficient of error (CE) was calculated following the formula:

(8) $$\begin{array}{cc} \text{CE} & ={\left[\frac{k}{k-1}\left\{\frac{\sum u^{2}}{\sum u\sum u}+\frac{\sum u^{2}}{\sum v\sum v}-2\frac{\sum uv}{\sum u\sum u}\right\}\right]}^{1/2}. \end{array}$$

In this formula, there are *k* images, and each summation is over 1 to *k*. An example of the application of this type of calculation is given in Table 1. The coefficient of error (CE) of this estimate was approximated using (8).

### 2.9. Statistical Analyses

The statistical analyses were performed using statistical package for the Social Sciences for Windows (SPSS, Inc., Chicago, IL) 7.5 version software. Mean values are presented with their standard deviations. We assessed the mean differences in the CV for 30 patients and 25 control subjects using independent sample Student's *t*-tests. Significance was indicated by a two-tailed *P* value of less than 0.05.

## 3. Results

There were 13 males and 12 females in the control group with a mean age of 39.24 ± 11.43. The mean (±SD) PCFV, CV, and CV to PCFV ratios were 165.57 ± 19.37 cm^3^, 125.74 ± 16.25 cm^3^, and 76.06 ± 6.54% in control subjects, respectively (Table 2). In males, the mean (±SD) PCFV, CV, and CV to PCFV ratios were 172.98 ± 20.36 cm^3^, 133.10 ± 15.29 cm^3^, and 77.35 ± 8.42%, respectively. In females, the mean (±SD) PCFV, CV, and CV to PCFV ratios were 157.54 ± 15.25 cm^3^, 117.77 ± 13.70 cm^3^, and 74.68 ± 3.47%, respectively. While there were no significant differences for age and CV to PCFV ratio (*P* < 0.05), a significant difference was found for PCFV and CV between genders (*P* > 0.05; Table 2).

There were 14 males and 16 females in the CMI group with a mean age of 39.63 ± 12.88. The mean (±SD) PCFV, CV, herniated tonsillar volume, herniated tonsillar length, CV to PCFV ratio, and herniated tonsillar volume to CV ratio were 146.01 ± 19.07 cm^3^, 117.49 ± 18.28 cm^3^, 0.89 ± 0.50 cm^3^, 9.63 ± 3.37 mm, 80.39 ± 6.68%, and 0.77 ± 0.38% in the CMI group, respectively (Table 3).

In males, the mean (±SD) PCFV, CV, herniated tonsillar volume, herniated tonsillar length, CV to PCFV ratio, and herniated tonsillar volume to CV ratio were 152.53 ± 24.79 cm^3^, 122.58 ± 21.50 cm^3^, 1.01 ± 0.61 cm^3^, 10.35 ± 3.93 mm, 80.39 ± 5.65%, and 0.83 ± 0.42%, respectively. In females, the mean (±SD) PCFV, CV, herniated tonsillar volume, herniated tonsillar length, CV to PCFV ratio, and herniated tonsillar volume to CV ratio were 140.24 ± 9.81 cm^3^, 113.03 ± 14.14 cm^3^, 0.79 ± 0.37 cm^3^, 9.00 ± 2.78 mm, 80.40 ± 7.65%, and 0.71 ± 0.35%, respectively. There were no significant differences between genders for all parameters (*P* > 0.05) (Table 3).

The PCFV in the CMI patients was significantly smaller than in the control subjects (*P* < 0.05). In the CMI group, the CV was smaller than in the control group, but this difference was not statistically significant (*P* > 0.05). In the CMI group, the CV to PCFV ratio was higher than in the control group. Our results revealed that chiari subjects had less PCFV and CV than the control group (Table 4).

**Figure 3:**
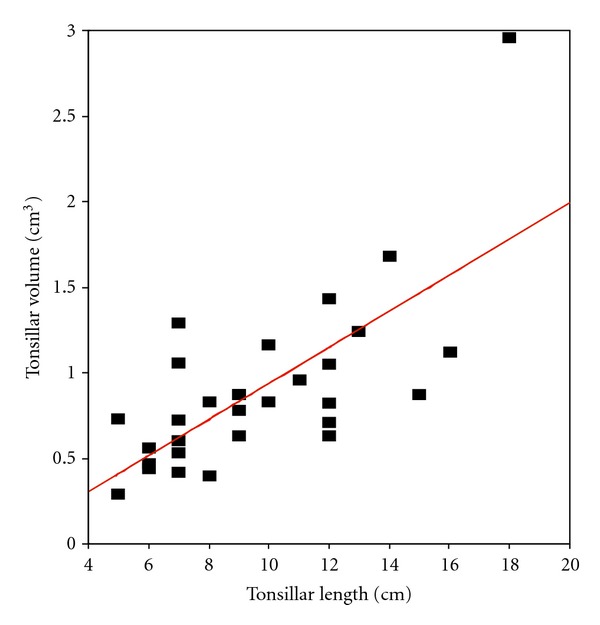
The figure shows the correlation between the herniated tonsillar length and volume.

There was a correlation between the PCFV and CV (*r* = 0.799, *P* < 0.001) in the control subjects and CMI group (*r* = 0.865, *P* < 0.001). There was also a correlation between herniated tonsillar volume and length (*r* = 0.703, *P* < 0.001) in the CMI group (Figure 3) (Table 5).

The mean time (±SD) needed to estimate the CV and herniated tonsillar volume using the point-counting technique was 5 ± 2.1 min with a range of 2–8 min. The mean of the CE for the estimation of herniated tonsillar volume to CV ratio was under 10%.

## 4. Discussion

Magnetic resonance imaging has become a useful diagnostic and investigative tool in brain research. Therefore, it is essential for the quantitative analysis of volumetric estimation. This quantitative information allows researchers to study the potential relationship between subtle neuroanatomic changes along with some neurological and neuropsychiatric diseases.

Several studies have found associations between cerebellar atrophy and neuropsychiatric symptomatology. The cerebellum is known to be involved in such diseases as alcoholism and ataxia [19, 20].

Stereological methods provide quantitative data on three-dimensional structures using two-dimensional images, although several studies have considered estimating the PCFV and cerebellar volume [7, 12, 17, 21]. According to our knowledge, there is no study on both cerebellar and herniated tonsillar volumes or CV to PCFV fraction that applies the unbiased techniques of stereological methods using MRI.

 The etiology of chiari type I malformation remains unclear. One theory to describe this form of hindbrain herniation suggests that a smaller than normal posterior cranial fossa predisposes a normal-sized cerebellum to traverse the foramen magnum during development [9].

Ekinci et al. [17] used the MRIs obtained from 24 normal volunteers ranging from 20 to 25 years of age and measured the total brain, cerebral, and cerebellar volume. They found that the mean cerebellar volume was 117.75 ± 10.7 cm^3^ and 111.83 ± 8.0 cm^3^ in males and females, respectively. Acer et al. [12] used MRI for CV estimates using two different methods in both sexes. They found that the mean results of the stereological method were 116.69 ± 10.1 cm^3^ and 114.41 ± 9.3 cm^3^ in males and females, respectively. Their results demonstrated that female subjects had smaller cerebellar volumes than males. However, the difference between the genders was not statistically significant (*P* > 0.05). We found that the mean (±SD) CV was 133.10 ± 15.29 cm^3^ and 117.77 ± 13.70 cm^3^ in the male and female control subjects, respectively. Our research yielded similar results to other previous research.

Milhorat et al. [9] found that the total volume of the posterior cranial fossa was decreased by an average of more than 10 cm^3^ in some patients with CMI compared with a control population. Tubbs et al. [7] found that the mean PCFV was 208.5 cm^3^ in chiari patients. They did not find any statistically significant difference between controls and patients with CMI.

Furtado et al. [22] found that the PCFV in childhood patients with chiari malformation was significantly lower than in the control group (*P* = 0.002). They found that the mean PCFV was 204.1 cm^3^ in patients with CMI and 252.8 cm^3^ in the age- and sex-matched control group. Also, they found that the PCFV in adult patients with chiari malformation was 245.4 cm^3^. Nishikawa et al. [23] found that the PCFV in adult chiari patients and the PCFV in the control group were 186 cm^3^ and 193 cm^3^, respectively. They noted a smaller PCFV in the CMI patients. Our results revealed that chiari subjects had less PCFV and CV than the control group.

Schady et al. [24] found an inverse relationship between the size of the posterior cranial fossa and the degree of cerebellar herniation, whereas Stovner et al. [25] showed a strong positive correlation. We found that there was a correlation between the PCFV and CV in the control and chiari groups in our study.

The chiari type I malformation is traditionally characterized by the downward herniation of the cerebellar tonsils with a descent of 5 mm or more below the foramen magnum [26]. In literature, in patients with chiari type I malformations, herniation of the cerebellar tonsils is within a range of 3 to 29 mm below the foramen magnum [3, 22, 26]. Our results are similiar to the values obtained by other authors, and we showed a correlation between herniated tonsillar volume and length in the chiari group.

## 5. Conclusion

This study has shown that there are statically significant differences in the posterior cranial fossa volumes between CMI patients and control subjects. On the other hand, smaller CV is seen in CMI patients, but this difference is not statistically significant. We have highlighted several new features, such as herniated tonsillar volume of the CMI malformation that provide for a better understanding of how to use it as a radiological assessment. We also found a positive correlation between the PCFV and CV for each group. There was also a correlation between herniated tonsillar volume and length in the CMI group.

We believe that these correlations and measurements will facilitate the diagnosis of chiari malformations by radiologists and neurosurgeons. The clinicians and radiologists can consider the size of the herniated tonsils of the cerebellum if they know the herniated tonsillar length. The findings of the current study using stereological methods provide useful data for the evaluation of normal and pathologic volumes of the cerebellar and posterior cranial fossa.

## Figures and Tables

**Table 1:** An example of the application of volume and the volume fraction method described in the present study. *P*(*Y*) = number of points hitting the cross section of the cerebellum. *P*(*X*) = number of points hitting the cross section of the herniated tonsil.

Section number	*P*(*Y*)	*P*(*X*)	*P*(*Y*) × *P*(*X*)	∑*P*(*Y*)^2^	∑*P*(*X*)^2^
1	2	0	0	4	0
2	6	7	42	36	49
3	13	9	117	169	81
4	20	19	380	400	361
5	21	16	336	441	256
6	24	15	360	576	225
7	30	14	420	900	196
8	29	16	464	841	256
9	29	13	377	841	169
10	23	12	276	529	144
11	21	11	231	441	121
12	27	10	270	729	100
13	26	11	286	676	121
14	22	9	198	484	81
15	20	6	120	400	36
16	20	3	60	400	9
17	17	1	17	289	1
18	10	1	10	100	1
19	7	0	0	49	0
20	6	0	0	36	0
21	2	0	0	4	0

Total	375	173	3964	8345	2207

**Table 2:** Mean (±SD) age, CV, PCFV, and CV to PCFV ratio for both sexes in the control group.

	Age	CV (cm^3^)	PCFV (cm^3^)	CV to PCFV ratio (%)
Men (*n*: 13)	38.30 ± 11.14	133.10 ± 15.29	172.98 ± 20.36	77.35 ± 8.42
Women (*n*: 12)	40.25 ± 12.14	117.77 ± 13.70	157.54 ± 15.23	74.68 ± 3.47
Total (*n*: 25)	39.24 ± 11.43	125.74 ± 16.25	165.57 ± 19.37	76.06 ± 6.54

*P*	0.681	0.015	0.440	0.319

CV: cerebellar volume, PCVF: posterior cranial fossa volume.

**Table 3:** Mean (±SD) values for age, CV, PCFV, and CV to PCFV ratio, herniated tonsillar length, herniated tonsillar volume, and herniated tonsillar volume to CV ratio for both sexes in the CMI group.

	Age	CV (cm^3^)	PCFV (cm^3^)	CV to PCFV ratio (%)	Herniated tonsil lenght (mm)	Herniated tonsil volume (cm^3^)	Herniated tonsil volume to CV ratio (%)
Men (*n*: 14)	37.0 ± 11.6	122.58 ± 21.50	152.53 ± 29.79	80.39 ± 5.65	10.35 ± 3.93	1.01 ± 0.61	0.83 ± 0.42
Women (*n*: 16)	41.9 ± 13.8	113.03 ± 14.14	140.24 ± 9.81	80.40 ± 7.65	9.0 ± 2.78	0.79 ± 0.37	0.71 ± 0.35
Total (*n*: 30)	39.63 ± 12.88	117.49 ± 18.28	146.01 ± 19.07	80.39 ± 6.68	9.63 ± 3.37	0.89 ± 0.50	0.77 ± 0.38

*P*	0.330	0.157	0.790	0.997	0.280	0.239	0.424

CV: cerebellar volume, PCVF: posterior cranial fossa volume.

**Table 4:** Comparison of CV, PCFV, and CV to PCFV ratio between the two groups.

	Control (*n* = 25) Mean ± SD	CMI (*n* = 30) Mean ± SD	*P*
CV (cm^3^)	125.74 ± 16.25	117.49 ± 18.28	0.085
PCFV (cm^3^)	165.57 ± 19.37	146.01 ± 19.07	0.001

CV to PCFV ratio (%)	76.06 ± 6.54	80.39 ± 6.68	0.019

CV: cerebellar volume, PCVF: posterior cranial fossa volume.

**Table 5:** Correlation values among the three parameters.

Parameters	Pearson correlation test	
	Correlation	Significance
PCFV-CV(Control)	0.799	*P* < 0.001
PCFV-CV (CMI)	0.865	*P* < 0.001
Herniated tonsillar length-herniated tonsillar volume	0.703	*P* < 0.001

CV: cerebellar volume, PCVF: posterior cranial fossa volume.
